# Integrative multi-omics analysis unveils the regulatory landscape of diabetic cardiomyopathy: from chromatin accessibility to transcript isoforms and epitranscriptome

**DOI:** 10.3389/fendo.2026.1928778

**Published:** 2026-09-16

**Authors:** Licheng Ding, Kaiyuan Liu, Xiaofeng Ge, Zhaokai Li, Ke Hu, Shichen Bu

**Affiliations:** 1 Department of Cardiology, Xiamen Cardiovascular Hospital of Xiamen University, School of Medicine, Fujian Branch of National Clinical Research Center for Cardiovascular Diseases, Xiamen, China; 2 Department of Vascular Surgery, Renmin Hospital of Wuhan University, Wuhan, China

**Keywords:** diabetic cardiomyopathy, epitranscriptomics, *Fam210b*, multi-omics integration, T-cell infiltration

## Abstract

Diabetic cardiomyopathy (DCM) is a major contributor to heart failure in diabetic patients, characterized by profound metabolic remodeling and diastolic dysfunction. However, the multi-layered epitranscriptomic and post-transcriptional networks involved in this disease remain poorly understood. To address this, we established a type 2 diabetes-associated DCM mouse model using a high-fat diet (HFD) combined with low-dose streptozotocin (STZ) injections. By integrating single-molecule direct RNA sequencing (DRS-seq), chromatin accessibility profiling (ATAC-seq), and conventional bulk RNA-seq, we constructed a comprehensive epigenetic-transcriptional regulatory map of DCM. Using DRS-seq, we identified 21,156 full-length transcripts, including 8,457 (39.97%) novel unannotated isoforms, and observed a systemic 3’-UTR elongation under diabetic stress. Joint ATAC-seq and DRS-seq analysis identified concurrent reductions in promoter chromatin accessibility and transcript abundance for four genes, including *Fam210b* (mitochondrial iron homeostasis), *Cdh22* (intercalated disc adhesion), *Fbxo10* (ubiquitin-mediated RAGE degradation), and *Cenpx* (DNA double-strand break repair), suggesting a potential link between altered chromatin accessibility and transcriptional regulation in DCM. Additionally, DRS-seq revealed extensive alterations in the cardiac epitranscriptome, identifying 1,719 differential m^6^A sites and 3,146 differential m^5^C sites at single-molecule resolution. Among these epitranscriptomic changes, we observed a potential post-transcriptional interplay between m^6^A and alternative polyadenylation (APA). Furthermore, by applying the CIBERSORT algorithm to the DRS-seq dataset, we characterized the cardiac immune microenvironment, revealing distinct pathological remodeling defined by decreased plasma cells and Th1 cells alongside a specific increase in CD4+ memory T-cell infiltration. Pearson correlation analysis showed that, among the candidate genes, only the down-regulated mitochondrial regulator *Fam210b* exhibited a significant negative correlation with CD4+ memory T-cell infiltration, suggesting a potential molecular association between mitochondrial dysfunction and local adaptive immune changes. Together, our study provides a high-resolution landscape of the native cardiac transcriptome and epitranscriptome, highlighting epigenetic-transcriptional coupling, m^6^A-APA cooperative decay, and *Fam210b*-associated local immune microenvironment remodeling as potential pathogenic contributor, thus offering candidate therapeutic targets for diabetic heart failure.

## 1 Introduction

Diabetic cardiomyopathy (DCM) is an insidious and progressive complication of type 2 diabetes, presenting clinically as myocardial hypertrophy, interstitial fibrosis, and early diastolic dysfunction that ultimately culminates in heart failure (1). Under chronic hyperglycemic and insulin-resistant conditions, the myocardium undergoes a profound metabolic substrate shift, characterized by metabolic inflexibility, an overreliance on fatty acid β-oxidation, lipotoxicity, oxidative stress, and mitochondrial decay (2). While epigenetic modifications and transcriptional networks have been extensively implicated in these pathological transitions, the post-transcriptional landscape (including full-length transcript isoforms, alternative polyadenylation (APA) dynamics, and the epitranscriptome) remains poorly defined in the diabetic heart. Specifically, how chromatin accessibility, splicing complexity, and chemical RNA modifications synergistically coordinate the early metabolic adaptation and late structural deterioration of the diabetic myocardium is still a fundamental unanswered question in cardiovascular medicine (3).

Traditional high-throughput bulk RNA sequencing (bulk RNA-seq) relies on complementary DNA (cDNA) synthesis and PCR amplification, which inherently introduce composition biases, underrepresent low-abundance transcripts, and completely erase native RNA chemical modifications (3). Consequently, bulk RNA-seq is inadequate for resolving the full complexity of splicing isoforms, multi-site methylation, and alternative polyadenylation (4). Single-molecule direct RNA sequencing (DRS-seq) has emerged as a revolutionary technology that bypasses reverse transcription and PCR entirely, thereby preserving the original molecular features of RNA (5). By directly reading native RNA translocating through synthetic nanopores, DRS-seq enables the unbiased identification of full-length transcripts, precise poly(A) tail length estimation, and the simultaneous, single-molecule resolution detection of epitranscriptomic modifications such as N^6^-methyladenosine (m^6^A) and 5-methylcytosine (m^5^C) (6). Crucially, because m^6^A deposition and APA are highly coordinated post-transcriptional processes that dictate mRNA stability, localization, and translation, investigating their synergistic crosstalk is essential to deciphering transcriptomic decay in diseased states.

In this study, we established a high-fat diet (HFD) and streptozotocin (STZ) -induced type 2 diabetic mouse model of DCM and integrated ATAC-seq, DRS-seq, and bulk RNA-seq to decode the multi-layered regulatory architecture of the diabetic myocardium. We identified 21,156 full-length transcripts, revealing that nearly 40% represent novel unannotated isoforms and that diabetic stress induces systematic 3’-untranslated region (3’-UTR) elongation, which compromises transcript stability. By linking promoter chromatin compaction with direct single-molecule transcript profiling, we identified a potential epigenetic-transcriptional coupling pattern associated with the suppression of key genes, including *Fam210b*, *Cdh22*, *Fbxo10*, and *Cenpx*, which are involved in mitochondrial dysfunction, cell adhesion failure, persistent inflammation, and genomic instability. Furthermore, we mapped the extensive dynamic remodeling of both m^6^A and m^5^C epitranscriptomes. Importantly, a subset of transcripts showed concurrent differential expression, altered APA site usage, and changes in m^6^A modification. Among these, *Hmgcs2*, *Fbxo21*, *Gadd45b*, and *Lipe*, which are involved in ketogenesis, ubiquitin-mediated proteolysis, the DNA damage response, and lipolysis, respectively, exhibited changes in both m^6^A modification and APA. These coincident alterations suggested a possible association between m^6^A status and APA processing, with potential consequences for transcript stability and functional output.

Beyond intrinsic cardiomyocyte metabolic and post-transcriptional dysfunction, the chronic inflammatory microenvironment is recognized as a key driver of cardiac remodeling in DCM (7). However, how specific cardiomyocyte epigenetic and epitranscriptomic decay events communicate with local immune cell populations remains largely unresolved. To explore this, we employed the CIBERSORT algorithm to deconvolve the myocardial immune landscape from DRS-seq profiles. This analysis indicated alterations in the myocardial immune microenvironment, characterized by reduced plasma cells and Th1 cells together with increased infiltration of CD4+ memory T cells. Integration with the transcriptional candidates further showed that, among the identified genes, the down-regulated mitochondrial gene *Fam210b* was significantly and negatively correlated with CD4+ memory T-cell infiltration, suggesting a potential association between altered myocardial mitochondrial regulation and local adaptive immune responses.

In conclusion, our work provides a high-resolution native epitranscriptomic and transcriptomic landscape of DCM, highlighting the m^6^A-APA interplay and *Fam210b*-associated immune microenvironment changes as potential post-transcriptional mechanisms in cardiac pathology. These findings offer a resource for future mechanistic and therapeutic investigations.

## 2 Materials and methods

### 2.1 Construction of a diabetic cardiomyopathy mouse model

The construction of the diabetic mouse model was performed as described above (8). All experimental mice used in this study were purchased from Shulaibao (Wuhan) Biotechnology Co., Ltd. (Wuhan, China). To establish a type 2 diabetes mellitus (T2DM) associated cardiomyopathy model, 7-week-old male C57BL/6J mice were first fed HFD for 8 weeks, followed by intraperitoneal injections of STZ at a dose of 35 mg/kg on 5 consecutive days. Age-matched control mice were maintained on a standard chow diet. Hyperglycemia was validated 1 week following the terminal STZ injection, with animals displaying random blood glucose thresholds of ≥16.7mmol/L designated as diabetic and subsequently sustained on HFD for an additional 8 weeks. All mice were then euthanized, and ventricular tissues were rapidly collected for subsequent experiments. Pentobarbital sodium via intraperitoneal injection was used for both euthanasia (200 mg/kg body weight) and anaesthesia (50 mg/kg body weight) in this study. Additionally, mice were lightly anaesthetized with 2% isoflurane inhalation prior to echocardiography.

### 2.2 RNA isolation and purity identification

Total RNA was extracted from tissue/cell samples using the RNA Easy Fast Tissue/Cell Kit (DP451, TIANGEN, China). Total RNA concentration and chemical purity were spectrophotometrically assessed by determining the A260/A280 absorbance ratio on an Implen N50 Touch NanoPhotometer (Implen GmbH, Munich, Germany). Intact RNA structural profile was verified by running the isolated samples on a 1.0% agarose gel.

### 2.3 RNA sequencing

For directional RNA-seq library synthesis, 1 μg of intact total RNA per biological replicate was used. Polyadenylated transcripts were captured using VAHTS mRNA Capture Magnetic Beads (Vazyme Biotech, N401), followed by genomic DNA elimination with RQ1 DNase (Promega). The purified mRNA fraction underwent thermal fragmentation and was subsequently reverse-transcribed into double-stranded cDNA. After end-repair, dA-tailing, and adapter ligation using the VAHTS RNA Multiplex Oligos Set 1 for Illumina (Vazyme, N323), size selection targeted library inserts of 300–500 bp. To preserve strand specificity, the dUTP-incorporated second strand was omitted from amplification. The final multiplexed libraries were quantified and stored at −80 °C prior to 150 nt paired-end sequencing on an DNBSEQ-T7 platform. Raw reads were filtered to remove those containing more than two ambiguous nucleotides (N). Adapter sequences and low-quality bases were trimmed using FASTX-Toolkit (v0.0.13), and reads shorter than 16 nt were excluded. Clean, high-quality reads were aligned against the mouse reference assembly (GRCm39) using HISAT2 (9), under a constraint of a maximum of four mismatches (≤4). To ensure maximum accuracy in downstream expression estimation, only uniquely aligned reads were retained for gene-level raw counting and subsequent transcripts per million (TPM) profiling.

### 2.4 Differentially expressed genes analysis

The R/Bioconductor tool DESeq2 (v1.30.1) was employed to identify transcriptional differences across cohorts (10). Transcripts were classified as significantly altered if they exhibited a fold change of ≥2 or ≤0.5 concurrently with an associated P-value < 0.05.

### 2.5 ATAC sequencing and data processing

Paired-end sequencing reads were aligned to the GRCm39 assembly using Bowtie2 (v2.4.4) under local, highly sensitive search constraints with strict fragment size filters (--local --very-sensitive --no-mixed --no-discordant -I 10 -X 700) to discard discordant and low-quality alignments. PCR duplicates were computationally pruned using sambamba (v0.8.1). Alignments on non-canonical chromosomes were excluded, and samtools (v1.7) was applied to retain uniquely mapped and multi-mapped populations. Upon coordinate sorting and indexing of BAM files using samtools (v1.7), peak calling was executed utilizing MACS2 (v2.2.7.1) with optimized parameters adapted to narrow marks (-f BAMPE --keep-dup all) or broad domains (--broad --broad-cutoff 0.1 -f BAMPE --keep-dup all). Processed BAM files were subsequently used for peak-region read density calculation. Enriched DNA-binding motif footprints were characterized using HOMER (11). Differential peak accessibility across conditions was resolved using DESeq2 (10), applying a statutory significance threshold of p < 0.05.

### 2.6 Library preparation, and direct RNA sequencing

Only samples with RNA integrity numbers (RINs) > 7 were selected for long-read DRS, as lower quality RNA was unlikely to yield informative results. Polyadenylated mRNA was purified from total RNA preparations utilizing the NEBNext Poly(A) mRNA Magnetic Isolation Module. Isolated poly(A)+ RNA was used for library preparation with the Direct RNA sequencing Kit (SQK-RNA004) protocol. The workflow comprised RT adapter ligation, reverse transcription and purification, sequencing adapter ligation and purification, and flow cell priming and loading. All surfaces and equipment were decontaminated with RNaseZAP before use. For RT adapter ligation, 300 ng of poly(A)-tailed RNA was diluted to 8 µl with nuclease-free water and combined with 3 µl NEBNext^®^ Quick Ligation Reaction Buffer (NEB, B6058), 1 µl murine RNase inhibitor (NEB, M0314), 1 µl RT adapter (ONT, SQK-RNA004), and 1.5 µl T4 DNA ligase (2 million U/ml; NEB, M0202M) to a total volume of 15 µl. The mixture was gently mixed, briefly centrifuged, and incubated at room temperature for 10 min. Reverse transcription was performed by adding a master mix of nuclease-free water, dNTPs (NEB, N0447), and Induro^®^ RT Reaction Buffer (NEB, B0681AVIAL), followed by Induro^®^ Reverse Transcriptase (NEB, M0681), and incubated at 60 °C for 30 min, then 70 °C for 10 min. The reverse transcription product was purified with Agencourt RNAClean XP beads (Beckman Coulter™, A63987) using 70% ethanol washes and eluted in 23 µl nuclease-free water. Sequencing adapter ligation was performed by mixing 23 µl cDNA, 8 µl NEBNext^®^ Quick Ligation Reaction Buffer, 6 µl RNA ligation adapter (ONT, SQK-RNA004), and 3 µl T4 DNA ligase, incubated for 10 min at room temperature. The ligation product was purified using Agencourt RNAClean XP beads with ONT Wash Buffer, followed by elution in RNA Elution Buffer (ONT, SQK-RNA004). Final libraries were quantified with Qubit fluorometry, targeting >30 ng recovery, and loaded onto flow cells following ONT priming and loading procedures, ensuring careful removal of air bubbles to preserve pore integrity and sequencing performance. All sequencing runs were performed on MinION flow cells. Sequencing runs were controlled via MinKNOW software (v 25.03.7) using the direct RNA protocol.

The raw data format of Nanopore sequencing off-machine data is pod5 format, which contains all raw sequencing signals, Dorado (version: 1.0.2) software was used to perform (Base Calling, inosine_m6A_2OmeA@v1, m6A_DRACH, m5C_2OmeC, --estimate-poly-a) analysis and converts pod5 format data into bam format, which contains the base information of sequencing sequences (reads), their corresponding sequencing quality information, methylation site and polyA length information.

Full-length transcript reads were recovered and trimmed with Pychopper (v2.7.9). Quality control was performed using Chopper (v0.7.0), retaining reads with a quality score ≥10 and length ≥50 bp. High-quality reads were mapped to the Ensembl GRCm39 genome using Minimap2 (v2.27-r1193) in splice-aware mode (-ax splice -uf -k14). To reconstruct and curate high-confidence full-length transcript models, the FLAIR computational pipeline was implemented for isoform assembly and stringent quality filtration (12). Candidate transcripts were retained if they satisfied the following multi-omic validation criteria: (1) 5′terminal positions were located within a 100-bp genomic window from transcription start sites (TSS) compiled in the refTSS6 database and/or classified by the TSS classifier tool (operating in relaxed or strict configurations) leveraging FANTOM CAGE expression peaks, and (2) isoforms were supported by at least three independent full-length reads (exhibiting >=80% alignment coverage and spanning a minimum of 25 bp of both terminal exons) evaluated via the FLAIR --stringent flag (12, 13). Subsequently, structural categorization and artifact identification were conducted using SQANTI3 under default algorithmic configurations to eliminate technical transcript chimeras (14).

### 2.7 Conversion of abundance estimates to transcripts per million

Salmon (v1.7.0) was run in quasi-mapping mode to estimate TPM values for individual transcripts and genes across all samples (12).

### 2.8 Differential gene and transcript expression, and transcript usage analysis

DESeq2 (v1.30.1) was applied to identify differentially expressed genes (DEGs) and transcripts (DTEs) between experimental groups. Statistical significance was defined as fold change ≥1.5 or ≤0.67 with p < 0.05 (10).

### 2.9 Alternative polyadenylation

Alternative polyadenylation (APA) events were characterized using TAPAS (15). Differential APA sites were identified with fold change ≥2 or ≤0.5 and false discovery rate (FDR) ≤ 0.05. APA-mediated transcript length changes were calculated as: lengthening = longer APA-shorter APA (negative logFC); shortening = shorter APA-longer APA (positive logFC). Motif discovery within 50 bp upstream of APA sites was conducted using the STREME algorithm (http://meme-suite.org/meme/tools).

### 2.10 Pathway enrichment analysis

Gene Ontology (GO) terms and Kyoto Encyclopedia of Genes and Genomes (KEGG) pathways were identified using KOBAS 2.09 Enrichment significance was determined by hypergeometric testing with Benjamini–Hochberg multiple-testing correction (16).

### 2.11 Immune cell infiltration deconvolution and Pearson correlation analysis

To accurately reconstruct the local immune cell infiltration microenvironment of the diabetic myocardium based on long-read direct RNA sequencing (DRS-seq) expression profiles, CIBERSORT, a linear support vector regression-based deconvolution framework, was applied. Non-log-transformed Transcripts Per Million (TPM) expression matrices obtained from DRS-seq were utilized as input (17). Deconvolution was performed using the mouse-specific LM22 signature gene expression matrix, which represents 22 distinct immune cell subtypes, including B cells, T-cell subsets, plasma cells, natural killer cells, and myeloid lineages. A total of 1,000 permutations were executed to estimate the relative proportions of immune cells, and only samples with a deconvolution significance of p<0.05 were retained. Pearson correlation analysis was subsequently performed to evaluate the relationship between the expression levels of eight candidate transcripts (including epigenetic-coupled genes *Fam210b*, *Cdh22*, *Fbxo10*, and *Cenpx*, and m^6^A-APA-regulated genes *Hmgcs2*, *Fbxo21*, *Gadd45b*, and *Lipe*) and the CIBERSORT-derived infiltration percentage of CD4+ memory T cells. Pearson correlation coefficients (r) and double-tailed P values were calculated using the cor and cor.test packages in R, with |r|>0.6 and p<0.05 set as the significance threshold.

### 2.12 Echocardiography

Following the establishment of DCM, mice were lightly anaesthetized with 2% inhaled isoflurane. Cardiac function was evaluated by transthoracic echocardiography at baseline and immediately before euthanasia using a VINNO 6 VET ultrasound system (VINNO Technology, China). Image acquisition and quantitative analysis were performed by investigators blinded to the experimental group assignments.

### 2.13 Histological assessments

Following euthanasia, mouse hearts were promptly excised and washed with ice-cold 1× phosphate-buffered saline (PBS). The hearts were then perfused with 10% KCl to induce diastolic arrest and fixed overnight in 4% paraformaldehyde. After paraffin embedding, the tissues were transversely sectioned at a thickness of 5 μm. Three to five sections from each heart were evaluated, with microscopic fields examined per section. Myocardial fibrosis was evaluated by Masson’s trichrome staining, whereas fluorescent wheat germ agglutinin (WGA; Sigma-Aldrich) labeling was used to outline individual cardiomyocytes for morphometric assessment. All staining procedures were conducted following the supplier’s protocols. Bright-field or fluorescence images were captured using an Olympus microscope (Olympus, Japan), as appropriate, and quantitatively analyzed with Fiji software (ImageJ; National Institutes of Health, Bethesda, MD, USA).

### 2.14 Reverse transcription quantitative PCR and ELISA

RNA extraction, cDNA synthesis, quantitative RT-PCR reactions were performed as described previously (18). Primer sequences are provided in **Supplementary Table 4**. Plasma concentrations of atrial natriuretic peptide (ANP) and B-type natriuretic peptide (BNP) were measured using commercially available mouse-specific ELISA kits (ANP, E-EL-M0166; BNP, E-EL-M0204; Elabscience Biotechnology Co., Ltd., Wuhan, China) according to the manufacturer’s instructions. The concentrations of ANP and BNP were calculated from the corresponding standard curves.

### 2.15 Statistical analysis

Quantitative datasets were analyzed and visualized using R software (v4.2.3) within the RStudio integrated development environment. Numeric metrics are expressed as mean ± standard error of the mean (SEM). Statistical significance of differences between two independent groups was evaluated via two-tailed unpaired Student’s t-test.

## 3 Results

### 3.1 DRS-seq identifies the full-length transcriptional landscape of DCM

To evaluate the pathogenesis of DCM, male murine cohorts were subjected to a dual-stimulus protocol comprising a HFD and low-dose STZ, utilizing age-matched, standard chow-fed counterparts as normal controls (19). Following confirmation of successful DCM model establishment via echocardiography and histological assessments (**Supplementary Figure 1**), heart tissues were harvested from the NC (n=3) and DCM (n=3) groups for integrated multi-omics analysis, encompassing DRS-seq (20), ATAC-seq, and bulk RNA-seq. The results showed that the overall gene expression levels measured by bulk RNA-seq were significantly lower than those detected by DRS-seq (**Supplementary Figure 2**). This discrepancy in expression is presumably driven by the inherent biases of traditional cDNA sequencing toward reverse transcription and PCR amplification, which often leave low-abundance transcripts or highly complex splice isoforms undetected (21). Conversely, DRS-seq directly sequences full-length single-molecule native RNA, enabling the capture of low-abundance genes and complex isoforms with superior sensitivity and minimal bias, thereby circumventing the quantitative deviation characteristic of conventional sequencing (22). DRS-seq analysis revealed that the number of detected genes and transcripts was overall comparable across all six myocardial samples from the NC and DCM groups, with no statistically significant differences observed between the groups (**Figure 1A**). At the transcript structural level, the average length of transcripts in the DCM group was significantly longer than that in the NC group (**Figure 1B**). This elongation may be associated with aberrant RNA processing events in DCM, such as 3’-UTR lengthening, which subsequently impacts mRNA stability and translation efficiency (23, 24). Based on the mouse reference genome, a total of 21,156 transcripts were identified via DRS-seq, which included 8,457 novel transcripts (accounting for 39.97%) that have not been annotated in existing databases (**Figure 1C**). Subsequently, the detected transcripts were classified and functionally annotated using the SQANTI3 standard pipeline: full-splice match (FSM) and incomplete-splice match (ISM) transcripts were categorized as known transcripts of known genes; novel in catalog (NIC), novel not in catalog (NNC), fusion, genic genomic, and genic intron transcripts were identified as novel splice isoforms of known genes; whereas antisense and intergenic transcripts were defined as completely novel transcription products derived from unannotated novel genes (**Figure 1D**) (25). Analysis of transcript multiplicity per gene (isoform multiplicity) indicated that the vast majority of genes in myocardial tissues yielded only a single transcript, and multi-isoform genes with transcript counts ≥4 were exclusively of known types. Global analysis revealed that the number of known transcripts associated with a single gene was significantly higher than that of newly identified transcripts (**Figure 1E**). The transcription start sites (TSSs) of both known and novel transcripts were extracted, and read density profiles were generated around the TSS regions. Both transcript categories exhibited clear enrichment of reads around their respective TSSs, supporting the consistency and reliability of TSS identification (**Figure 1F**). Classification of transcripts according to functional type showed that protein-coding transcripts accounted for 90.6% of all detected transcripts (**Figure 1G**), indicating that protein-coding transcripts constituted the predominant transcript category in our dataset.

**Figure 1:**
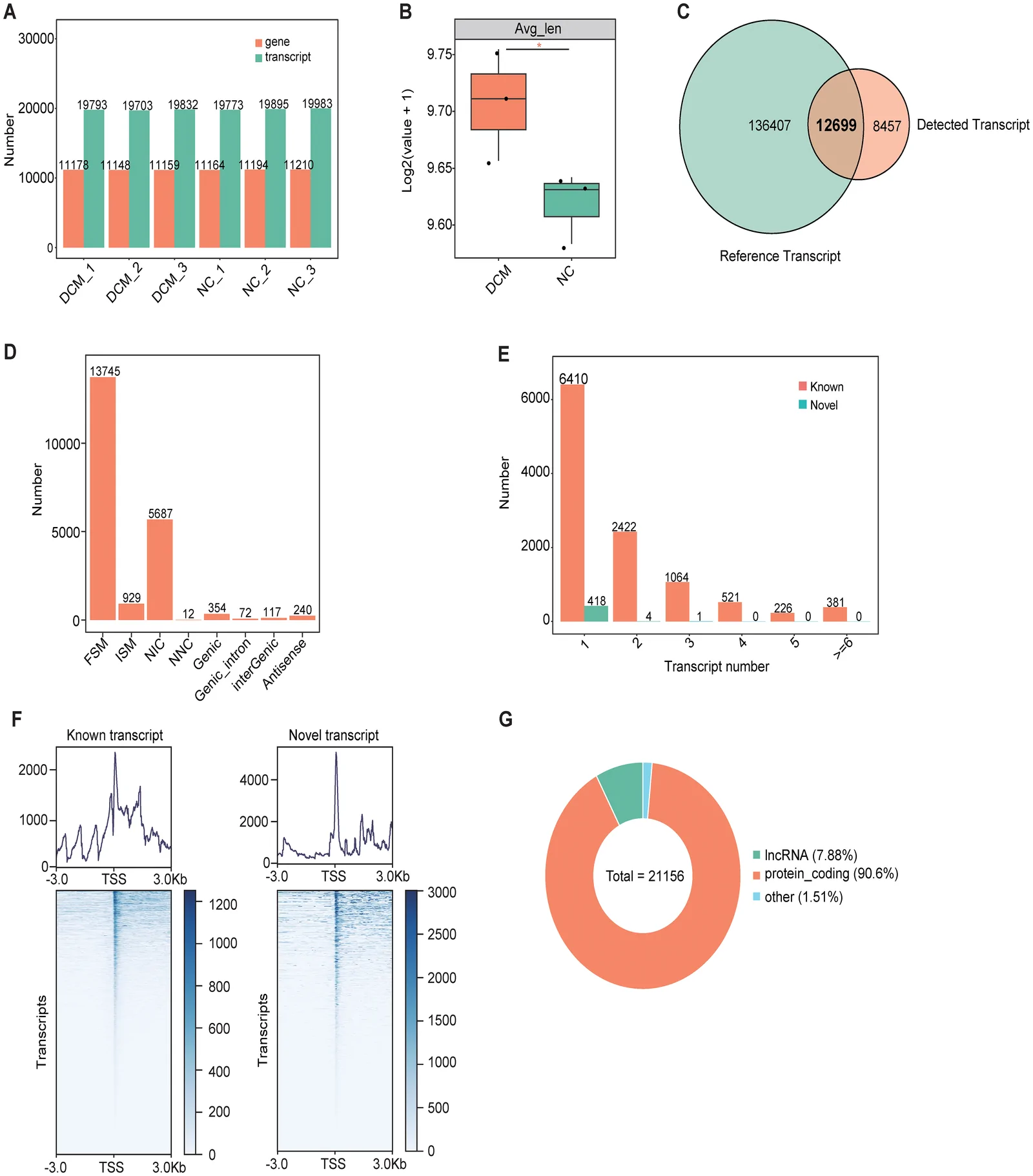
Identification and characterization of full-length transcripts in diabetic cardiomyopathy. **(A)** The bar showing the number of the transcripts and genes detected in all samples. **(B)** The boxplot showing the lengths of all sample transcripts. **(C)** The Venn diagram of the overlapping transcripts between reference genome transcripts and the number of detected transcripts. **(D)** The bar showing the number of transcript types. **(E)** The number of transcripts of single genes. **(F)** This figure shows the distribution of reads 3kb upstream and downstream of a gene transcription start site (TSS). **(G)** The percentage of the number of transcript types.

### 3.2 Multi-level transcriptomic profiling via DRS-seq reveals isoform-specific regulation and metabolic reprogramming in DCM

DRS-seq was employed to quantify the expression abundance of detected full-length transcripts, and the DESeq2 algorithm identified 607 differentially expressed transcripts (DETs), comprising 490 annotated and 117 novel unannotated transcripts (**Figure 2A**). GO-BP enrichment analysis of these 490 annotated DETs revealed significant enrichment in key pathways associated with myocardial fatty acid metabolism, including lipid metabolism, fatty acid β-oxidation, acyl-CoA dehydrogenase-dependent fatty acid β-oxidation, and fatty acid catabolism (**Supplementary Figure 3A**). Furthermore, functional enrichment prediction for the 117 novel unannotated transcripts also strongly implicated energy metabolic networks, such as lipid and fatty acid metabolism (**Figure 2B**), suggesting that these novel splice variants may act as pivotal post-transcriptional regulators participating in myocardial metabolic reprogramming in DCM (26). To dissect the pathogenic genes of DCM at a multidimensional transcriptomic level, differential expression analysis was first conducted at the gene level. The results showed that DRS-seq detected 440 differentially expressed genes (DEGs), whereas bulk RNA-sequencing (bulk RNA-seq) identified only 289 DEGs (**Figure 2C**). An intersection analysis of the DEGs identified by both platforms revealed that 81 core genes exhibited consistent and significant differential expression trends in both DRS-seq and bulk RNA-seq (**Figure 2D**). GO functional enrichment analysis of these 81 shared DEGs indicated that they were also significantly enriched in myocardial fatty acid metabolism, lipid metabolism, fatty acid β-oxidation, and long-chain fatty acid (LCFA) metabolic pathways (**Supplementary Figure 3B**). These findings collectively indicate that myocardial energy substrate metabolic dysfunction is a central pathophysiological hallmark of DCM progression (27). Subsequently, we analyzed the association between the abundance detection profiles of the DETs and DEGs identified by DRS-seq, revealing a total of 402 genes co-detected at both the transcript (DET) and gene (DEG) levels (**Figure 2E**). Quantitative analysis of average transcript isoform diversity per gene showed that genes differentially expressed exclusively at the DEG level possessed a significantly higher average number of transcripts than those differentially expressed solely at the DET level and those shared between DETs and DEGs (**Figure 2F**). Classification of transcript biotypes indicated that protein-coding mRNAs predominated among both platform-specific and shared DETs/DEGs (**Figure 2G**). This feature confirms that multi-level transcription and alternative splicing events under DCM pathology directly reshape the myocardial proteomic profile. To further investigate the distinct pathological functions of transcription events across different dimensions, GO and KEGG pathway enrichment analyses were conducted separately on three core gene sets (shared DET/DEG, DET-specific, and DEG-specific genes) (**Figures 2H, I**). Functional annotation of the overlapping DETs/DEGs revealed predominant enrichment in myocardial energy metabolic pathways centered on fatty acid β-oxidation and lipid metabolic networks. DET-specific genes were significantly enriched in cellular homeostatic mechanisms, such as proteostasis, intracellular cholesterol balance, and programmed cell death, whereas DEG-specific genes were enriched in diverse intracellular and transmembrane signaling pathways, including cytokine-mediated signal transduction. Taken together, these results characterized metabolic and transcriptomic alterations in DCM using DRS-seq and provide single-molecule-level evidence of isoform-specific regulatory changes associated with myocardial protein quality control (PQC), endoplasmic reticulum stress, and apoptosis. These findings suggested that full-length transcript isoform regulation may contribute to the post-transcriptional responses accompanying DCM progression.

**Figure 2:**
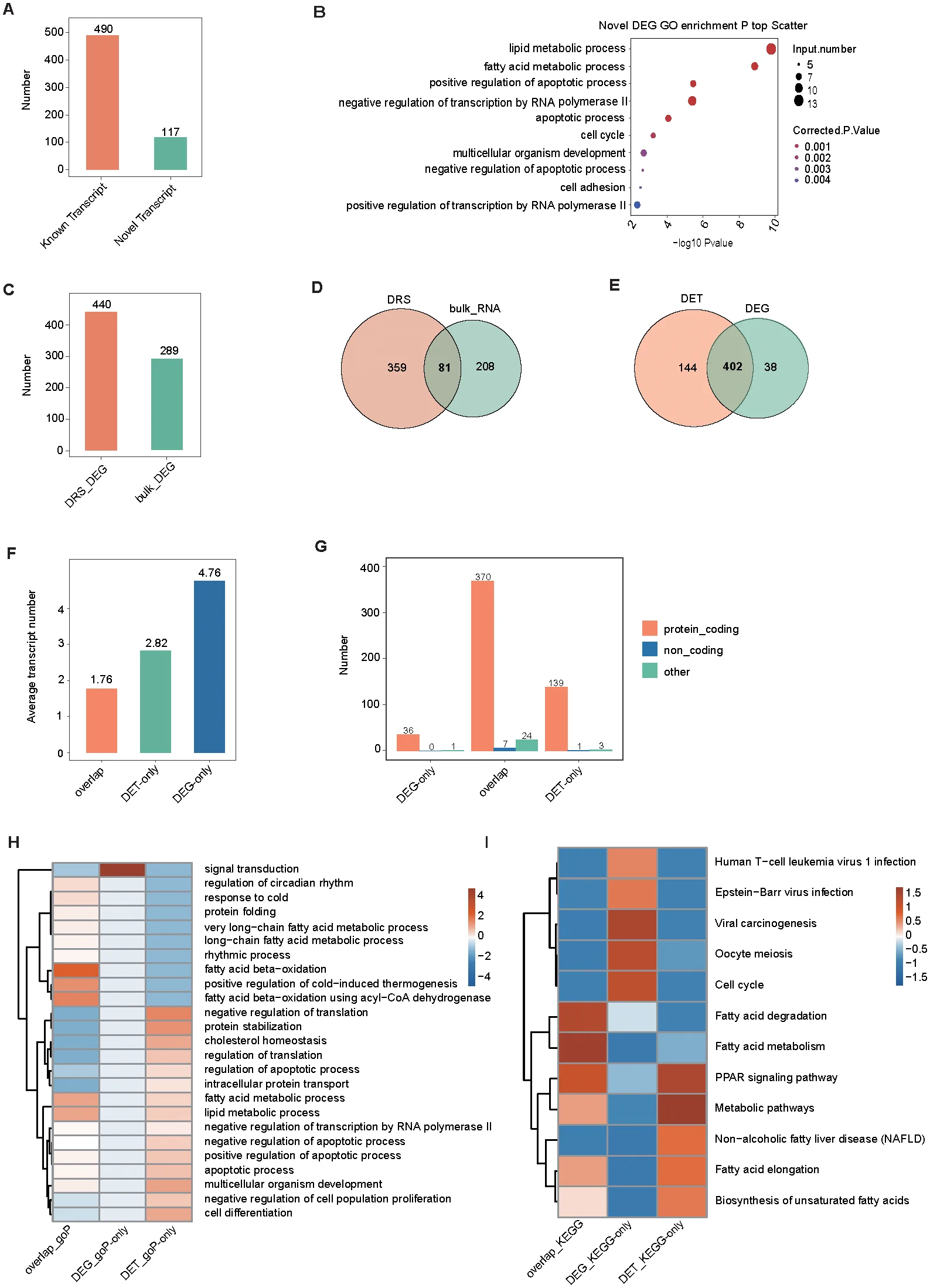
Functional annotation of differentially expressed transcripts (DET) in diabetic cardiomyopathy. **(A)** The bar showing the number of differentially expressed transcripts (DETs) based on known and novel. **(B)** The Scatter plot exhibiting the most enriched GO biological process results of the novel DETs. **(C)** The bar showing the number of differentially expressed genes (DEGs) for DRS-seq and bulk RNA-seq. **(D)** The Venn diagram of the overlapping DEGs between DRS-seq and bulk RNA-seq. **(E)** The Venn diagram of the overlapping genes between differentially expressed transcripts (DETs) and differentially expressed genes (DEGs). **(F)** The bar showing the average number of the average number of transcripts for each overlapping part. **(G)** The bar showing the number of gene types for each overlapping part. **(H)** Hierarchical clustering heat map showing the top enriched GO biological process results of the gene for each overlapping part. **(I)** Hierarchical clustering heat map showing the top enriched KEGG pathway results of the gene for each overlapping part.

### 3.3 Integrated ATAC-seq and DRS-seq profiling reveals epigenetic-transcriptional coupling in DCM

Assay for ATAC-seq is a pivotal epigenomic approach for analyzing physical chromatin accessibility and cis-regulatory element activity (28). To elucidate the molecular pathogenic mechanisms of DCM, this study integrated ATAC-seq and DRS-seq to systematically analyze the epigenetic-transcriptional coupling regulatory network in DCM. ATAC-seq analysis revealed that, compared with the NC group, a total of 71 up-regulated differentially accessible regions (up-DARs) with significantly increased accessibility and 547 down-regulated DARs (down-DARs) with decreased accessibility were identified in DCM (**Supplementary Figure 4A**). Compared with up-DARs, down-DARs exhibited a more pronounced enrichment pattern within genomic promoter regions (**Supplementary Figure 4B**). Downstream bioinformatic annotation demonstrated that the genes associated with promoter-localized up-DARs were primarily enriched in GO-BP such as multicellular organismal development; in KEGG pathways, they were significantly enriched in the Wnt signaling pathway and sphingolipid metabolism (**Supplementary Figure 4C**). Conversely, genes associated with promoter-localized down-DARs were significantly enriched in GO-BP terms related to the DNA damage response, double-strand break repair, intracellular protein transport, basic metabolism, negative regulation of the MAPK cascade, the upregulation of intracellular protein catabolism and the positive modulation of RNA polymerase II-dependent gene transcription. KEGG pathway analysis showed that these genes were markedly clustered in cellular senescence, the AMPK signaling pathway, the renin-angiotensin system (RAS), and the phosphatidylinositol signaling system (**Supplementary Figure 4D**). To identify differentially expressed transcripts (DETs) directly regulated by chromatin accessibility states, DETs identified via DRS-seq were integrated with promoter-associated DARs from ATAC-seq, screening out 15 candidate DETs potentially subject to epigenetic-transcriptional coupling regulation (**Figure 3A**). Among the 15 candidate molecules, specific transcripts of four genes exhibited concordant epigenetic regulation and transcriptional suppression, characterized by concurrent significant down-regulation of both transcript abundance and promoter chromatin accessibility; these genes were *Fam210b* (ENSMUST00000028995.5), *Cdh22* (ENSMUST00000065438.13), *Fbxo10* (ENSMUST00000052236.13), and *Cenpx* (e9369d06-d741-4a06-ac21-59fb2b0186cf). The expression levels of these four transcripts were verified by RT-qPCR (**Supplementary Figures 4E–H**). Meanwhile, all of these transcripts were cross-validated through read visualization analysis of conventional bulk RNA-seq data. This concordance identified these genes as candidates whose expression may be associated with altered chromatin state (**Figures 3B–E**). The synergistic down-regulation of these four key candidate transcripts may collectively mediate the transcriptional remodeling process during progressive myocardial injury in DCM by respectively disrupting mitochondrial energy remodeling and iron/heme homeostasis (*Fam210b*) (29), cardiomyocyte adhesion and mechanical structural homeostasis (*Cdh22*) (30, 31), ubiquitin-proteasome-mediated degradation quality control of pro-inflammatory receptors (*Fbxo10*) (32), and DNA damage repair and genomic stability maintenance (*Cenpx*) (33). In summary, the above results suggested *Fam210b*, *Cdh22*, *Fbxo10*, and *Cenpx* may serve as core target genes regulated by epigenetic modifications in DCM, offering novel epigenomic insights for further dissecting the transcriptional remodeling and pathological mechanisms underlying DCM.

**Figure 3:**
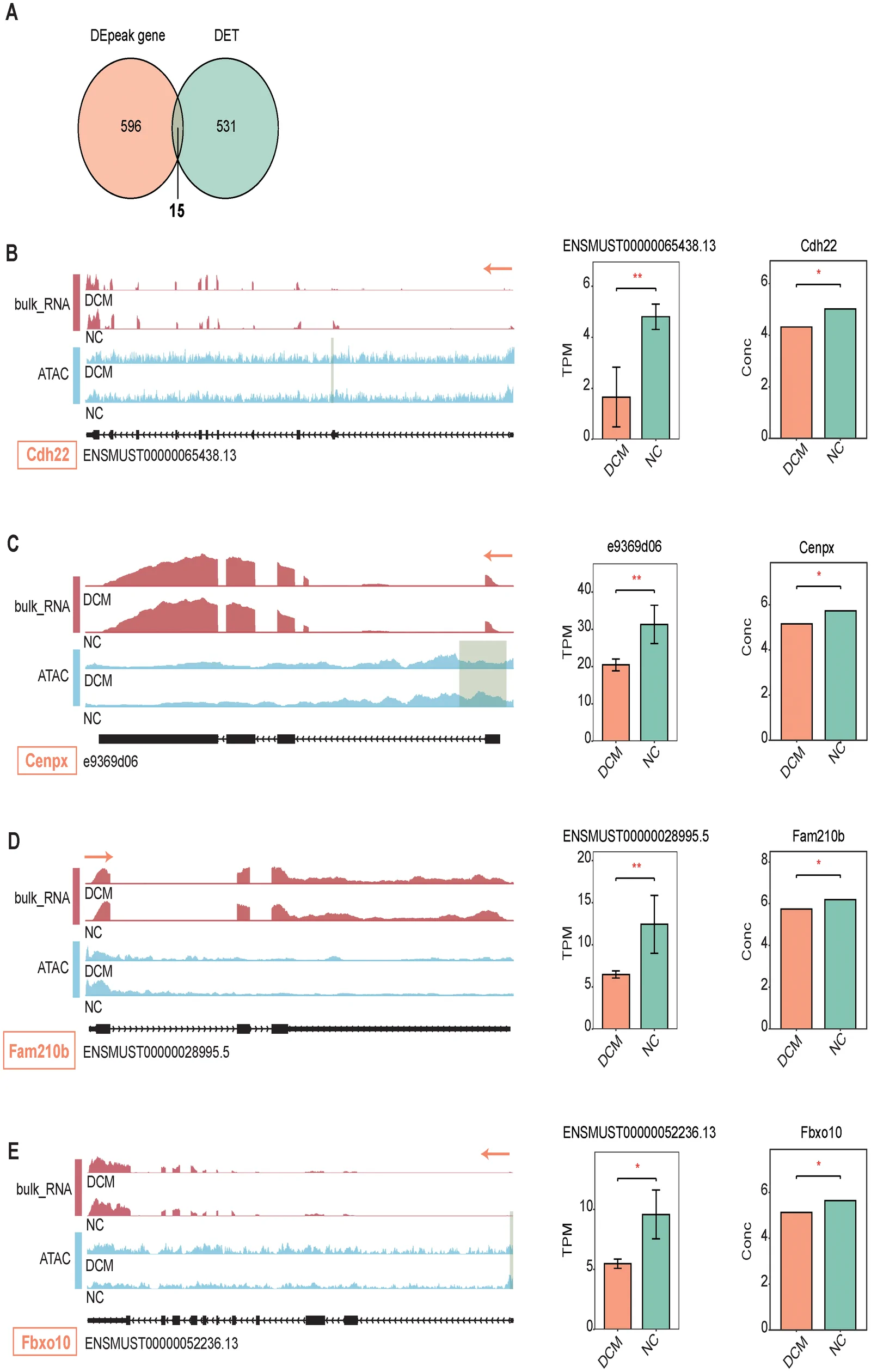
Integration of DET (DRS-seq) and DAR (ATAC-seq) of diabetic cardiomyopathy. **(A)** Venn diagram showing the DET of DAR. **(B–E)** Showing the transcripts information from overlapped transcripts (Fam210b, Cdh22, Fbxo10, Cenpx). Left panel: Schematic representation of distinct transcript isoforms identified by DRS-seq (black), along with read coverage from ATAC-seq (blue) and bulk RNA-seq (red). Right panel: Bar plot showing the relative expression levels of individual transcripts in DRS-seq and chromatin accessibility changes in ATAC-seq. Error bars represent mean ± SEM, **P-value < 0.01, *P < 0.05.

### 3.4 DRS-Seq reveals m^6^A and m^5^C methylation reprogramming in DCM

As a prevalent and highly conserved post-transcriptional modification in eukaryotic mRNAs, m^6^A plays a pivotal role in regulating key stages of gene expression, including RNA splicing, nucleocytoplasmic transport, transcript stability, and translation efficiency (34). Based on DRS-seq analysis, the total number of identified m^6^A modification sites in the DCM group was slightly reduced compared to the NC group, with approximately half of these sites exhibiting high methylation occupancy (m^6^A modification stoichiometry > 0.5%) (**Figure 4A**). Moreover, the vast majority of modified transcripts harbored an average of no fewer than 10 candidate m^6^A sites at the full-length level (**Figure 4B**), suggesting that their methylation modifications are characterized by highly coordinated multi-site regulation. Regarding transcriptomic topological distribution, these m^6^A sites spanned from the 5’-UTR to the 3’-UTR, with prominent enrichment observed near the stop codon of the coding sequence (CDS) and its adjacent exonic regions (**Figure 4C**). Specifically, the spatial distribution pattern of m^6^A remained highly consistent between the NC and DCM groups, characterized by primary enrichment in the 3’-UTR, followed by the CDS region, and extremely low abundance within the 5’-UTR. This conserved pattern indicates that under diabetic pathological stimuli, the global distribution characteristics of m^6^A modifications maintain a high degree of evolutionary or pathological conservation (**Figure 4D**). Differential methylation analysis identified a total of 1,719 differentially methylated sites (DMSs). Compared to the NC group, 746 sites in the DCM group exhibited significantly upregulated methylation levels, whereas 973 sites were significantly downregulated (**Figure 4E**). Notably, leveraging the high sensitivity of DRS-seq in capturing ionic current signal deviations, this study also identified widespread m^5^C modifications. Quantitative analysis revealed that healthy control displayed a slightly lower abundance of global m^5^C sites relative to the DCM group, and the majority of modified sites in both groups exhibited a relatively low methylation occupancy of below 0.5 (**Figure 4F**). Further analysis indicated that most target transcripts harbored an average of at least 15 candidate m^5^C modification sites at the single-molecule full-length level (**Figure 4G**). Transcriptomic regional localization demonstrated that these m^5^C sites widely spanned from the 5’-UTR to the 3’-UTR, with a predominant enrichment flanking the start and stop codons of the CDS, displaying a characteristic bimodal enrichment profile along the transcripts (**Figure 4H**). Inter-group comparisons of distribution abundance showed that m^5^C modifications were primarily enriched in the CDS region, followed by the 3’-UTR in both the NC and DCM groups, suggesting that their global topological features also exhibit a high degree of pathological conservation (**Figure 4I**). Differential methylation analysis identified a total of 3,146 differentially methylated m^5^C sites; compared to the NC group, 1,633 sites in the DCM group showed significantly upregulated modification levels, while 1,513 sites were significantly downregulated (**Figure 4J**). In summary, DRS-seq analysis identified alterations in m^6^A and m^5^C RNA methylation patterns in the diabetic myocardium. These findings suggested that changes in the RNA methylation landscape, together with potential interactions between different modification types, may be associated with the molecular alterations underlying DCM.

**Figure 4:**
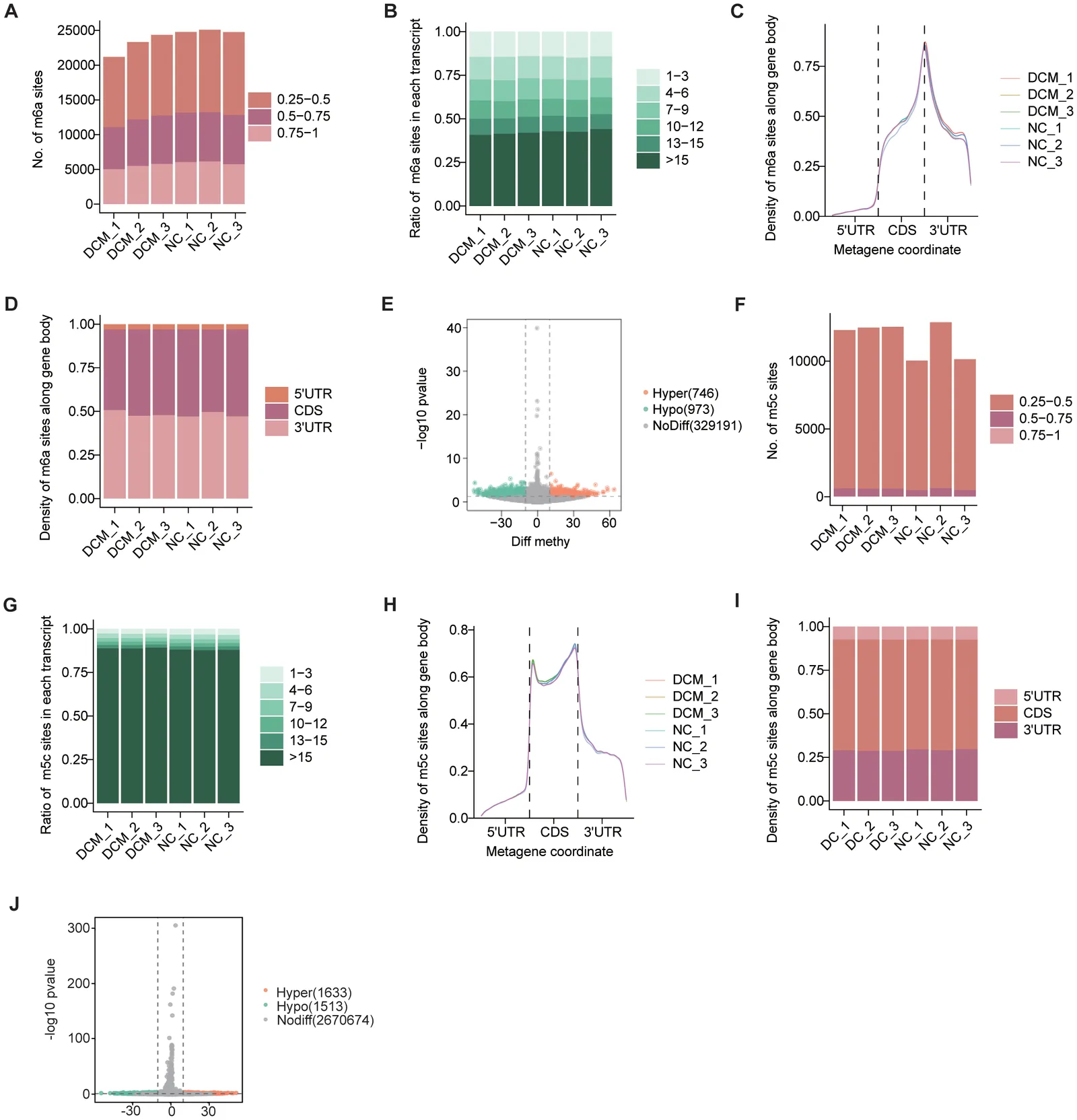
m6A modification of transcripts in DRS-seq. **(A)** The bar showing the number of m6A modified sites in all samples. **(B)** The ratio of transcripts with a different number of m6A-modified sites. **(C)** The density of m6A-modified bases along the gene body. The position of each modified site along the gene body was normalized by the length of the transcript using R pipeline MeraPlotR. **(D)** The ratio of m6A-modified sites distributed in the 5’-UTR, CDS,3’-UTR in all samples. **E)** Volcano plot showing all differentially expressed transcripts with a different number of m6A-modified sites (DETs). **(F)** The bar showing the number of m5C modified sites in all samples. **(G)** The ratio of transcripts with a different number of m5C-modified sites. **(H)** The density of m5C-modified bases along the gene body. The position of each modified site along the gene body was normalized by the length of the transcript using R pipeline MeraPlotR. **(I)** The ratio of m5C-modified sites distributed in the 5’-UTR, CDS,3’-UTR in all samples. **(J)** Volcano plot showing all differentially expressed transcripts with a different number of m5C-modified sites (DETs).

### 3.5 Cooperative post-transcriptional regulation by m^6^A and APA contributes DCM pathogenesis

Polyadenylation is a co-transcriptional process that covalently attaches a poly(A) tail to the 3’ ends of eukaryotic mRNAs. This modification serves multiple essential functions, including protecting transcripts from exoribonucleolytic degradation, ensuring proper transcription termination, and promoting the nucleocytoplasmic export of mRNAs (23). Alternative polyadenylation (APA) is a widespread post-transcriptional mechanism that generates transcript isoforms with distinct 3’-UTR lengths or coding sequences from a single gene locus. In this study, we employed DRS-seq to systematically identify and comprehensively characterize transcript polyadenylation sites (PAS). Quantitative transcripts characterization revealed that transcripts containing multiple alternative polyadenylation sites (≥4 PAS) were markedly depleted in the DCM cohort compared with the NC group (**Supplementary Figure 5A**), which directly indicating a systemic decline in myocardial transcript isoform complexity under pathological conditions. A total of 335 significantly shifted PAS were identified by inter-group comparative analysis (**Supplementary Figure 5B**). Following the reconstruction of 3’-UTR lengths based on these differential sites, a total of 320 transcripts were captured that underwent significant 3’-UTR lengthening or shortening events in the DCM group (**Supplementary Figure 5C**). Concurrently, profiling of global poly(A) tail lengths demonstrated that transcripts with lengths distributed around 50 bp were specifically decreased in DCM myocardial tissues (**Supplementary Figure 5D**). The differential analysis resolved a total of 276 differential poly(A) tail length events. Notably, the proportion of novel transcripts represented in these differential events significantly exceeded that of known transcripts (**Supplementary Figure 5E**), suggesting that poly(A) tail reprogramming may serve as a pivotal molecular foundation for novel transcripts to execute their gain-of-function mechanisms.

Subsequently, to systematically elucidate the synergistic interactions among multiple post-transcriptional regulatory layers in DCM, we performed a multidimensional overlap analysis (**Figure 5A**) of DETs, differentially m^6^A-modified transcripts (DEm^6^A), transcripts with altered poly(A) tail lengths (DEpoly(A)), and transcripts undergoing APA (DEAPA) identified by DRS-seq. Within the overlap of differentially expressed transcripts (DETs), DEpoly(A), and DEAPA events, there was one core gene whose transcripts simultaneously exhibited abundance variations, aberrant tail lengths, and APA events. Within the intersection of DETs, DEm^6^A, and DEpoly(A), we identified 5 genes whose transcripts harbored concurrent expression imbalance, aberrant tail lengths, and altered m^6^A methylation states. Crucially, within the intersection of DETs, differentially DEm^6^A, and DEAPA, a total of 12 transcripts were enriched, which simultaneously exhibited expression changes, APA site shifting, and pathological remodeling of m^6^A modification. Among these 12 highly co-regulated molecules, the aberrant expression of *Hmgcs2* (ENSMUST00000090746.3) (35), *Fbxo21* (ENSMUST00000035579.10) (36, 37), *Gadd45b* (ENSMUST00000015456.10) (38), and *Lip*e (ENSMUST00000054301.14) (39) was speculated to arise from the depletion of m^6^A modification on their transcripts(**Figures 5B–E**). The expression level of the four transcripts were verified by using RT-qPCR (**Supplementary Figures 5F–I**). This pattern suggests that RNA-modification status and 3’ end processing may undergo coordinated remodeling in DCM. Given that these target genes are involved in key myocardial metabolic and stress-response pathways, these findings suggested that the coordinated dysregulation of m^6^A methylation and APA may contribute to alterations in myocardial gene expression programs and phenotypic remodeling in DCM.

**Figure 5:**
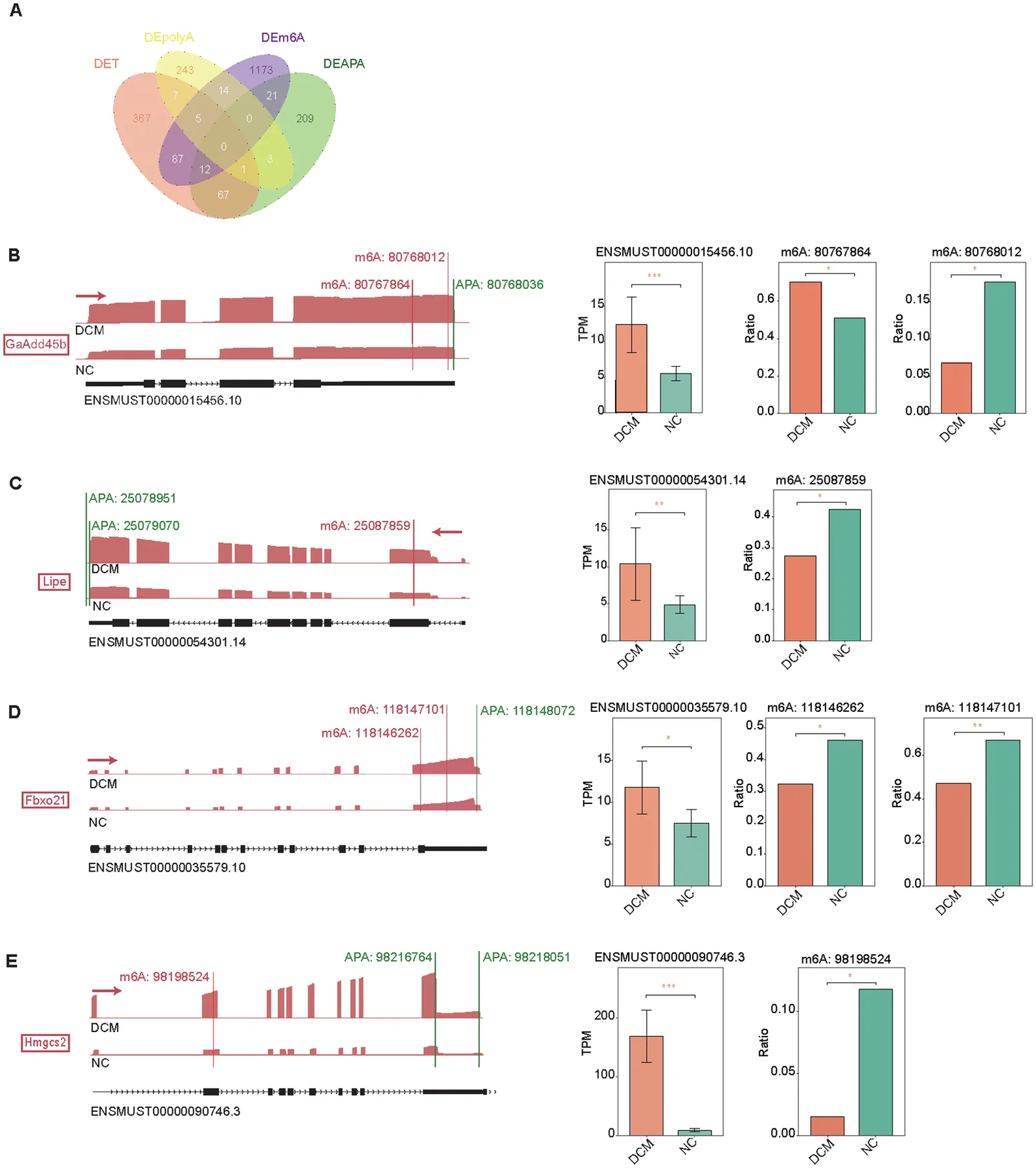
Integrative analysis of DETs, differentially m6A-modified transcripts, and alternative polyadenylation /polyA sites in DRS-seq. **(A)** Venn diagram showing the overlap of DET and DE-m6A and DE-polyA and DE-APA. **(B–E)** Showing the ENSMUST00000015456.10 of Gadd45b, ENSMUST00000054301.14 of Lipe, ENSMUST00000035579.10 of Fbxo21, ENSMUST00000090746.3 of Hmgcs2. Top panel: Reads distribution of the transcripts, full structure display, and distribution of APA and m^6^A sites. Bottom panel: Bar plot showing the expression levels of DETs and the modification ratios of m⁶A sites. Error bars represent mean ± SEM. **P-value < 0.01, *P < 0.05, ***P-value < 0.001.

### 3.6 Downregulation of mitochondrial *Fam210b* correlates with CD4+ memory T-cell infiltration and immune remodeling in DCM

To elucidate the immune cell infiltration profile and its potential impact on the transcriptional regulation of DCM, this study performed immune infiltration deconvolution analysis on DRS-seq data using the CIBERSORT algorithm (17). Computational deconvolution suggested a potential remodeling of the cardiac immune microenvironment in DCM mice, characterized by relatively reduced proportions of plasma cells and Th1 cells alongside a concomitant increase in CD4^+^ memory T-cell infiltration signals (**Figure 6A**). To identify key molecules mediating myocardial injury and local immune remodeling, we further performed correlation analyses between the infiltration levels of CD4+ memory T cells and eight candidate transcripts (*Fam210b*, *Cdh22*, *Fbxo10*, *Cenpx*, *Hmgcs2*, *Fbxo21*, *Gadd45b*, and *Lipe*) previously identified to exhibit epitranscriptomic-transcriptional co-suppression or m^6^A-APA-related regulation. The results indicated that among these eight genes, seven (*Fam210b*, *Cdh22*, *Fbxo10*, *Hmgcs2*, *Fbxo21*, *Gadd45b*, and *Lipe*) were identified as DEGs in the DRS-seq dataset (**Figure 6B**). Pearson correlation analysis demonstrated that among these seven DEGs, only *Fam210b* expression exhibited a statistically significant negative correlation with the proportion of infiltrating CD4+ memory T cells (Pearson r = -0.84, p < 0.05, **Figure 6C**). This finding suggested a potential link between *Fam210b* downregulation and the possible recruitment/activation of CD4^+^ memory T cells, hinting at a role for *Fam210b* in bridging myocardial mitochondrial dysfunction and local adaptive immune remodeling in DCM. Nevertheless, this mechanistic interpretation remains speculative and requires experimental substantiation (40).

**Figure 6:**
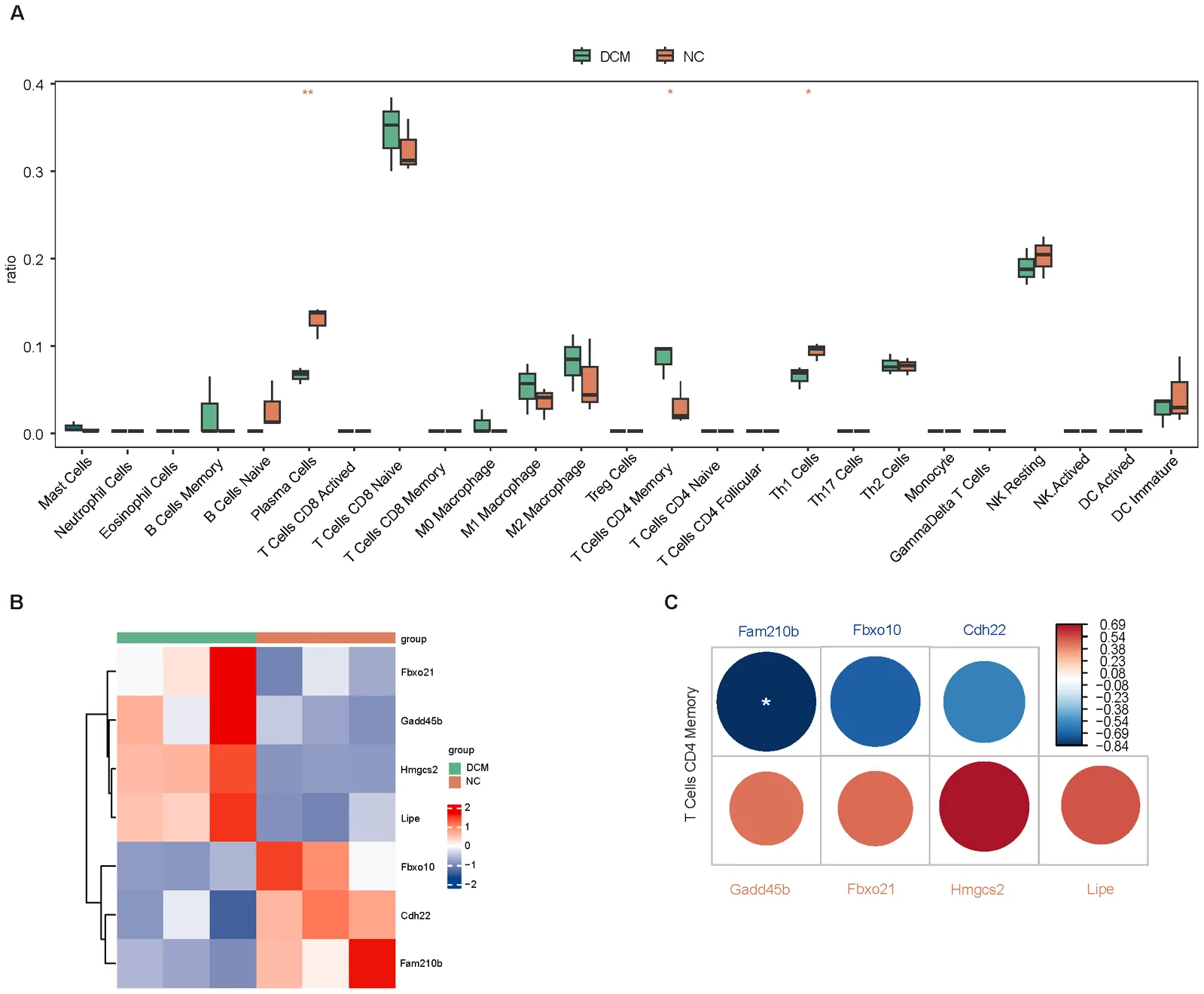
Fam210b correlates with CD4+ memory T-cell infiltration in DCM. **(A)** CIBERSORT analysis of cardiac immune microenvironment. **(B)** Verification of candidate transcript expression in DRS-seq datasets. **(C)** Pearson correlation matrix between candidate transcript expressions and CD4+ memory T-cell infiltration. **P-value < 0.01, *P < 0.05.

## 4 Discussion

In this study, we established a murine DCM model using long-term HFD exposure combined with low-dose STZ and applied integrated DRS-seq, ATAC-seq, and bulk RNA-seq to characterize regulatory alterations in the diabetic myocardium. Applying the SQANTI3 standard pipeline to the DRS-seq data resolved 21,156 transcripts, including 8,457 novel transcripts (39.97% of the detected transcriptome) that were unannotated in existing databases (25). At the structural level, a significant elongation of the average transcript length was observed in the DCM compared to healthy controls. This trend was accompanied by alterations in RNA processing, particularly 3′-UTR lengthening. Longer 3’-UTR are enriched with miRNA target sites and RBP binding motifs (41). Under chronic diabetic stress, such structural alterations may influence mRNA stability and translational regulation, suggesting that altered transcript architecture represents an additional layer of post-transcriptional dysregulation in DCM. In the present study, we identified a substantial number of previously unannotated transcript isoforms using DRS-seq. Nevertheless, independent validation of representative novel isoforms using Sanger sequencing or other complementary approaches will be important in future studies.

The healthy myocardium exhibits high metabolic flexibility, using both glucose and fatty acids (FAs) to sustain contractile function (42). In the diabetic state, insulin resistance and lipid excess force a severe fuel shift, leading to metabolic inflexibility and an overreliance on fatty acid β-oxidation (43). This metabolic reprogramming is a central hallmark of DCM, driving lipotoxicity and mitochondrial damage (44, 45). DRS-seq analysis identified 607 DETs, composed of 490 annotated and 117 novel unannotated transcripts. Enrichment analysis of both transcript classes highlighted myocardial energy metabolism, especially fatty acid metabolism, lipid metabolic processes, and acyl-CoA dehydrogenase-dependent fatty acid β-oxidation. This indicates that novel unannotated isoforms actively participate in the metabolic shifts of the diabetic heart. At the gene level, intersection analysis of the DEGs identified by DRS-seq and bulk RNA-seq revealed a core set of 81 shared DEGs with consistent and highly significant expression trends across both platforms. Functional enrichment of these shared core DEGs also converged on fatty acid and lipid metabolic pathways. This transcript-to-gene congruence demonstrates that energy metabolic substrate shifts are the dominant drivers of the cardiac transcriptional profile in DCM (46). Further analysis resolved 402 genes co-detected at both the DET and DEG levels. Notably, DEG-only genes, reflecting altered total gene abundance without detectable isoform switching, showed a higher average transcript number per locus than DET-specific or shared genes. This suggests that gene-level expression changes preferentially occur at complex multi-isoform loci, implying multilayered transcriptional and post-transcriptional regulation under diabetic stress. Functional separation of these gene sets showed that while shared DETs/DEGs coordinate energy metabolism, DET-specific genes are enriched in protein stabilization, intracellular cholesterol balance, and apoptosis. Thus, isoform-specific regulation may serve as a post-transcriptional buffer that supports cellular homeostasis, PQC, and cell survival under lipotoxic stress.

To determine how chromatin states control transcription in DCM, we integrated ATAC-seq with DRS-seq. ATAC-seq identified 71 up-DARs and 547 down-DARs. These down-DARs were highly enriched at promoters, suggesting broad epigenetic silencing of essential genes. Genes with promoter down-DARs were enriched in pathways like basic metabolism, cellular senescence, AMPK signaling, and the renin-angiotensin system (RAS). These pathways form key cytoprotective and metabolic networks that preserve cardiomyocyte viability under chronic stress (47). Simultaneously, AMPK acts as a metabolic sensor for mitochondrial quality control, and its impairment promotes mitochondrial decay, while overactive RAS drives adverse remodeling (48). Epigenetic silencing of these pathways suggests a collapse of baseline defense networks, accelerating DCM progression. Integrating promoter DARs with DRS-seq expression data identified 15 DETs potentially under direct epigenetic–transcriptional coupling. Among these, transcripts of four core genes—*Fam210b*, *Cdh22*, *Fbxo10*, and *Cenpx*—showed significant downregulation in both expression and promoter accessibility. *Fam210b* encodes a mitochondrial membrane protein required for iron import and heme biosynthesis, interacting with ATP synthase subunits and protoporphyrinogen oxidase (49). Silencing *Fam210b* reduces oxygen consumption and ATP production, disrupts iron sensing, and drives mitochondrial decay and ferroptotic cell death under diabetic lipotoxicity (29). In cardiomyocytes, the mechanical and electrical coupling occurs at intercalated discs via cadherins (50). CDH22 downregulation is predicted to disrupt disc integrity, impair mechanical coupling, and contribute to ventricular dilation and stiffness in the diabetic heart. FBXO10, part of the SCF E3 ubiquitin ligase complex, mediates clearance of BCL2 and targets the receptor for advanced glycation end products (RAGE) for degradation (32, 51). We hypothesize that compaction of the *Fbxo10* promoter blocks RAGE degradation, leading to its pathological accumulation. Finally, CENPX (MHF2) is part of the Fanconi anemia complex that stabilizes stalled replication forks and repairs DNA interstrand cross-links (33). We hypothesize that silencing *Cenpx* impairs DNA repair, leading to genomic instability, persistent DNA damage response activation, and accelerated cardiomyocyte senescence or apoptosis. However, the concurrent reduction in promoter accessibility and transcript abundance does not establish a causal relationship between these two events. Further experimental validation is therefore required to determine whether altered chromatin accessibility contributes directly to transcriptional dysregulation in DCM.

The m^6^A and m^5^C are key post-transcriptional marks regulating mRNA splicing, localization, stability, and translation (52). DRS-seq can simultaneously detect m^6^A and m^5^C modifications at single-molecule resolution (53). In DCM, total m^6^A sites were slightly reduced, with ~50% showing high occupancy (>0.5). Topologically, m^6^A was enriched near stop codons and in 3’-UTRs in both groups. However, we found 1,719 differentially methylated m^6^A sites in DCM (746 up, 973 down). This dynamic remodeling permits precise transcript-level regulation. The m^6^A modifications have been shown to modulate myocardial fibrosis, hypertrophy, and pyroptosis in DCM. For example, METTL14 promotes m^6^A modification of NLRP3 to aggravate pyroptosis in DCM, while YTHDF2-driven RMRP degradation inhibits it (54–56). Similarly, m^5^C modifications were widely detected, with bimodal enrichment flanking the start and stop codons of the CDS. A total of 3,146 differential m^5^C sites were identified in DCM. The m^5^C reader Alyref has been implicated in the orchestration of post-infarction myocardial remodeling through the selective control of extracellular matrix (ECM) protein synthesis in cardiac fibroblasts (60). Accumulating evidence highlights m^5^C methylation as a critical regulator of cardiac homeostasis and pathological remodeling. The methyltransferase NSUN2 was shown to promote pathological cardiac hypertrophy and heart failure by methylating the La-related protein (LARP1) transcript, thereby stabilizing GATA4 mRNA (57). Additionally, myofibroblast-specific depletion of NSUN2 has been shown to alleviate cardiac fibrosis post-myocardial infarction by reducing the stability of HuR transcripts (58).Nevertheless, the differential m^6^A and m^5^C sites identified by DRS-seq remain to be independently validated using conventional modification-specific assays. Further experimental validation of representative sites will be necessary to confirm the reliability of these epitranscriptomic changes and to clarify their potential functional relevance in DCM.

Polyadenylation at the 3’ end is critical for transcript stability, poly(A) tail length, and mRNA export. APA determines 3’-UTR length, affecting miRNA/RBP binding (41). DRS-seq showed fewer genes with ≥4 PAS in DCM, suggesting a systemic decline in transcript isoform complexity. Multidimensional overlap analysis of DETs, DEm^6^A, DEpoly(A), and DEAPA identified 12 transcripts undergoing concurrent expression changes, APA shifting, and m^6^A remodeling. Among these, we identified four key regulators (*Hmgcs2*, *Fbxo21*, *Gadd45b*, *Lipe*) whose upregulation is linked to reduced m^6^A. Increasing evidence suggests a high degree of coordination between m^6^A modification and APA. For example, the m^6^A reader IGF2BP2 binds to the APA factor CPSF6 to regulate 3’ end processing (59). And the depletion of m^6^A modifications on the transcripts disrupts the recruitment of the cleavage and polyadenylation specificity factor (CPSF) complex (such as NUDT21 or CPSF6) (60). This may block proper cleavage at proximal PAS, causing read-through and abnormal 3’ end formation. Thus, dual m^6^A-APA defects are proposed to disrupt expression of these key genes. HMGCS2 is the rate-limiting enzyme in ketogenesis; its down-regulation impairs myocardial ketone body utilization (35). FBXO21 is crucial for ubiquitin-mediated proteasomal degradation; its dysregulation compromises PQC (36, 37). GADD45B mediates the DNA damage response and cell cycle control; its silencing restricts DNA repair (61). LIPE (hormone-sensitive lipase) maintains lipolytic homeostasis; its impairment leads to lipid accumulation and cardiac lipotoxicity (62). It should be acknowledged that our multi-omic overlap analysis is inherently limited in establishing temporal or causal relationships. Whether the proposed mechanism indeed plays a role in DCM thus warrants further investigation.

Recent studies have shown that chronic hyperglycemia induces aberrant adaptive immune activation in the myocardium, recruiting peripheral immune cells (63). These infiltrating immune cells engage in complex interactions with cardiomyocytes and fibroblasts. By releasing pro-inflammatory and pro-fibrotic cytokines, they directly drive excessive extracellular matrix (ECM) deposition and myocardial stiffening (64, 65). This process is considered one of the primary driving force propelling early compensatory myocardial hypertrophy toward end-stage decompensated heart failure (66). In this study, DCM hearts showed fewer resident plasma cells and Th1 cells, but significantly more CD4^+^ memory T cells. CD4^+^ memory T cells respond rapidly to antigens or stress and secrete inflammatory cytokines (67). During the pathological progression of type 2 diabetes, an increased proportion of CD4+ memory T cells are closely associated with low-grade inflammation and exacerbated insulin resistance (68). Of all candidate targets in this study, only *Fam210b* exhibited a prominent negative correlation with the abundance of infiltrated CD4+ memory T cells. Previous studies have found that approximately 15.7% of homozygous *Fam210b* knockout (*Fam210b*−/−) mice spontaneously develop a systemic autoimmune phenotype resembling severe systemic lupus erythematosus(SLE) (69). Another study showed that *Fam210b* loss causes excess ROS in hematopoietic cells, breaking immune tolerance and triggering autoimmunity (29). Under diabetic metabolic stress, the promoter region of *Fam210b* may exhibit reduced chromatin accessibility accompanied by transcriptional repression. We further speculate that decreased *Fam210b* expression may contribute to mitochondrial dysfunction in cardiomyocytes, whereas its potential effects on mitochondrial iron homeostasis and oxidative stress remain to be experimentally determined. Recent studies have also confirmed that FAM210B can directly interact with the mitochondrial outer membrane translocase TOM70, and that the integrity of this complex plays a crucial regulatory role in maintaining mitochondrial innate immune feedback (70). Our correlation analysis supports a potential association between reduced *Fam210b* expression and increased CD4^+^ memory T-cell infiltration in DCM. However, these findings are primarily based on transcriptomic deconvolution and correlative expression analyses and therefore require further experimental validation.

In conclusion, our integrated multi-omics study provides a comprehensive map of the native transcriptomic, epitranscriptomic, and immune-infiltrating landscapes in DCM. By linking physical chromatin compaction with direct single-molecule RNA profiling, we identified several candidate genes and pathways potentially involved in DCM pathology: (1) epigenetic-associated suppression of *Fam210b*, *Cdh22*, *Fbxo10*, and *Cenpx*; (2) m^6^A-APA interplay potentially affecting metabolic regulators such as *Hmgcs2*, *Fbxo21*, *Gadd45b*, and *Lipe*; and (3) a distinct immune microenvironment with increased CD4+ memory T cells, which correlates with reduced *Fam210b* expression. These molecular alterations and changes in immune cell infiltration may represent potentially relevant features of DCM and warrant further investigation for their biological and translational significance. Further studies are needed to clarify the role of *Fam210b* in mitochondrial dysfunction and myocardial immune remodeling and to determine whether it has potential translational relevance in DCM. Overall, several limitations must be acknowledged. A primary limitation of this study is the modest sample size of the animal cohorts (n=3 per group), which potentially curtails the statistical power required to resolve highly dynamic or low-abundance transcriptional changes. Second, our sequencing was performed on bulk myocardial tissue, potentially masking cell-type-specific isoform heterogeneities, epitranscriptomic dynamics, and immune cell deconvolution resolution within cardiac fibroblasts, endothelial cells, or infiltrating immune populations. Third, diastolic function and broader metabolic phenotyping were not assessed, representing a limitation of the current DCM model characterization that warrants further investigation. Finally, while the HFD/STZ mouse model reproduces several key features of diabetic cardiomyopathy, a translational gap remains between rodent models and the long-term, multi-organ clinical complexity of type 2 diabetes in human patients. Future studies using single-cell long-read sequencing and human failing heart biopsies are warranted to validate these post-transcriptional and immunometabolism regulatory networks.

## Data Availability

The datasets presented in this study can be found in online repositories. The names of the repository/repositories and accession number(s) can be found in the article/**Supplementary Material**.
