# Supplementary material for: Integrative multi-omics analysis unveils the regulatory landscape of diabetic cardiomyopathy: from chromatin accessibility to transcript isoforms and epitranscriptome
Source: Front Endocrinol (Lausanne). 2026 Sep 16;17:1928778. doi: 10.3389/fendo.2026.1928778 (PMC13623679; doi:10.3389/fendo.2026.1928778)
Supplement: Supplementary file 1 [file Presentation1.pdf]

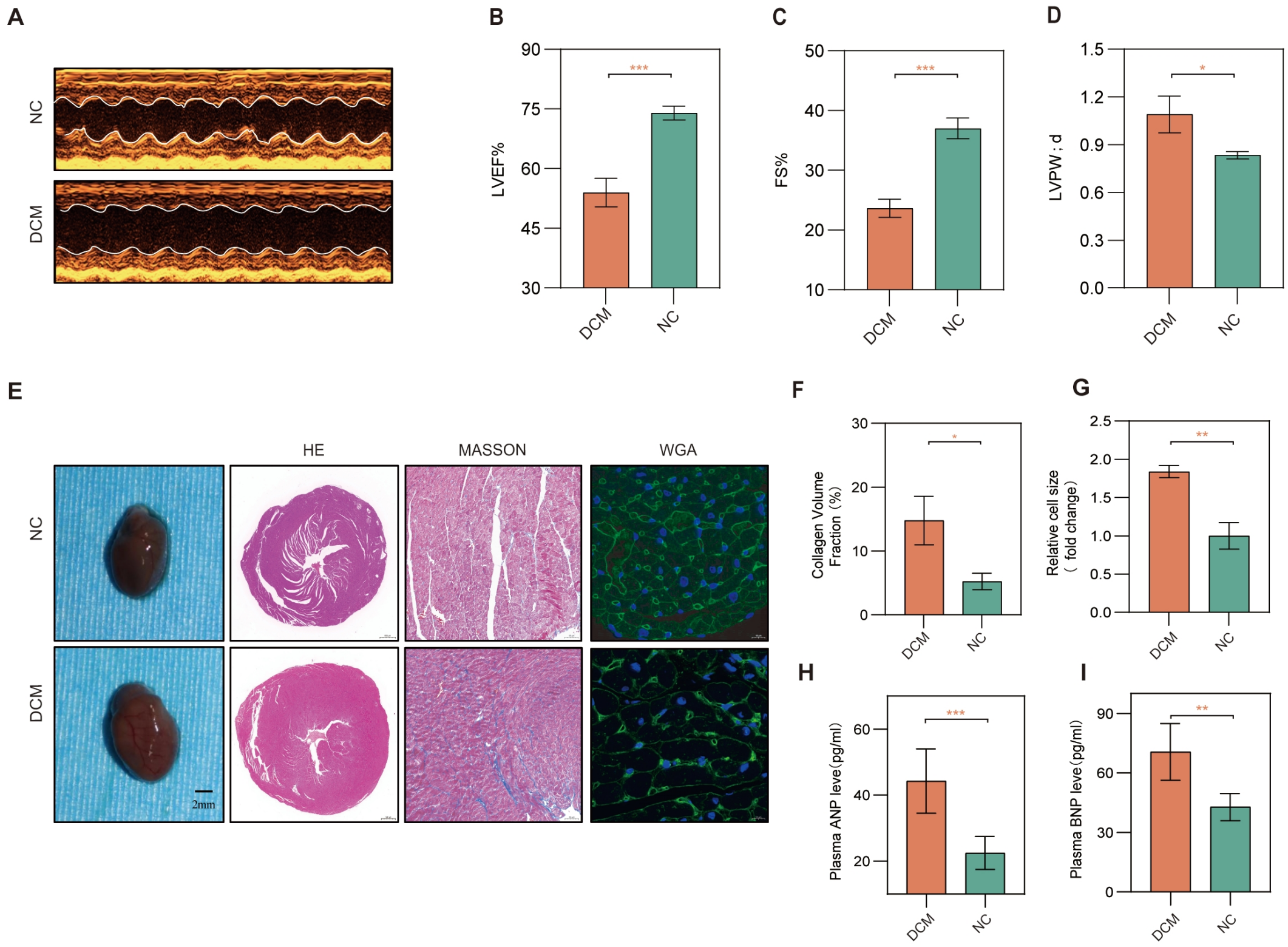

Figure S1. Characterization of a Mouse Model of Diabetic Cardiomyopathy

A-D. Echocardiographic parameters, including ejection fraction (EF) and fractional shortening (FS), were measured in DCM mice and non-diabetic controls (n = 3 per group; representative data are shown in panels B and C).

E-G. Representative images of whole hearts, along with HE-, Masson-, and WGA-stained myocardial sections, are presented, accompanied by the quantified data for interstitial fibrosis (F) and myocyte size (G) (n = 3 per group).

H-I. Plasma levels of ANP (atrial natriuretic peptide) and BNP (brain natriuretic peptide) were evaluated, with n = 3 per group. Error bars represent mean ± SEM, ***P-value＜0.001，**P-value < 0.01, *P < 0.05.

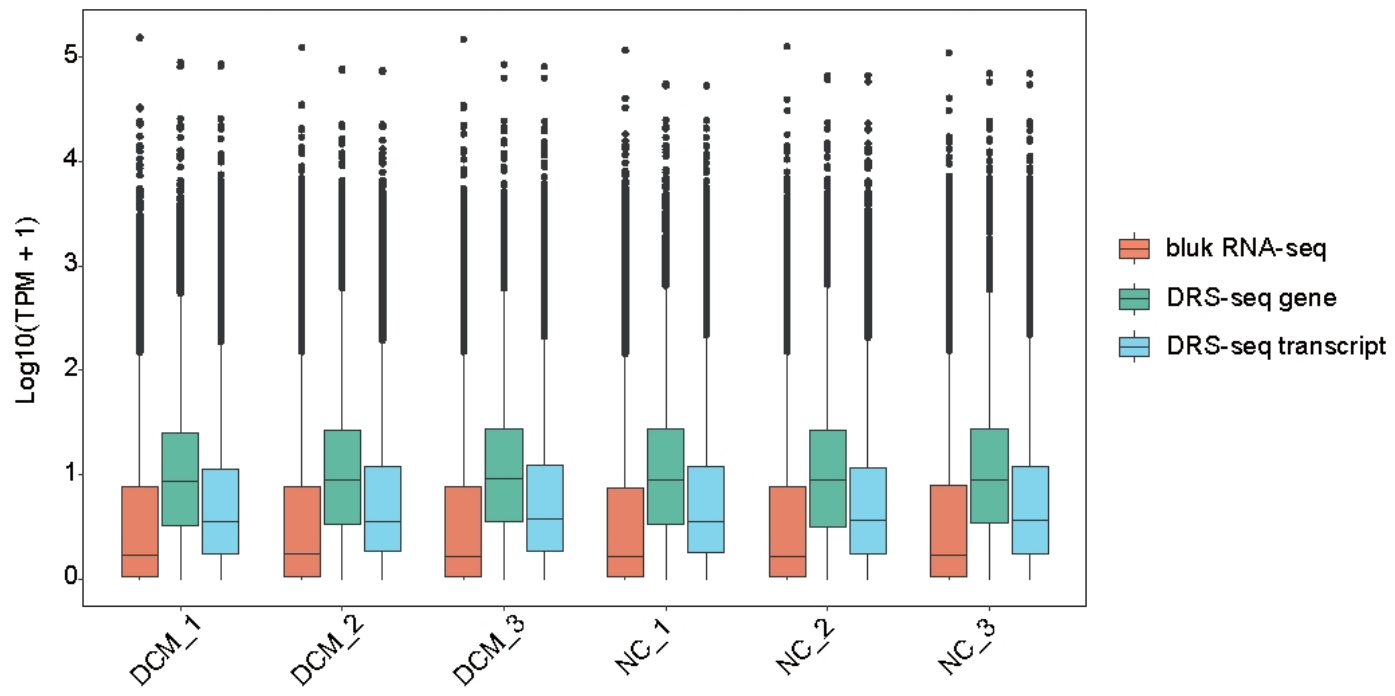

Figure S2. Analysis of gene and transcript expression abundance.

The boxplot showing the expression levels of genes and transcripts of DRS-seq, bulk RNA-seq.

A

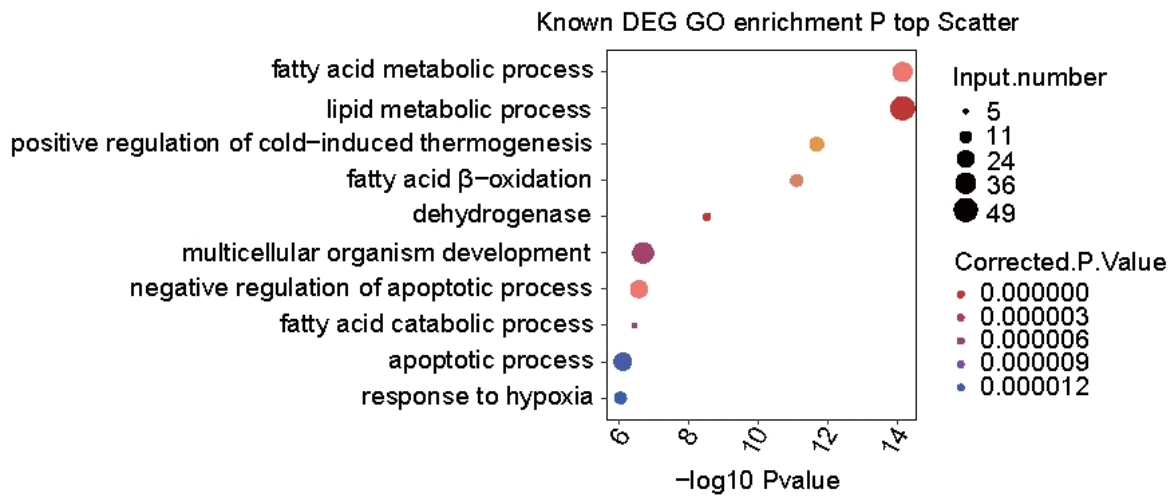

B

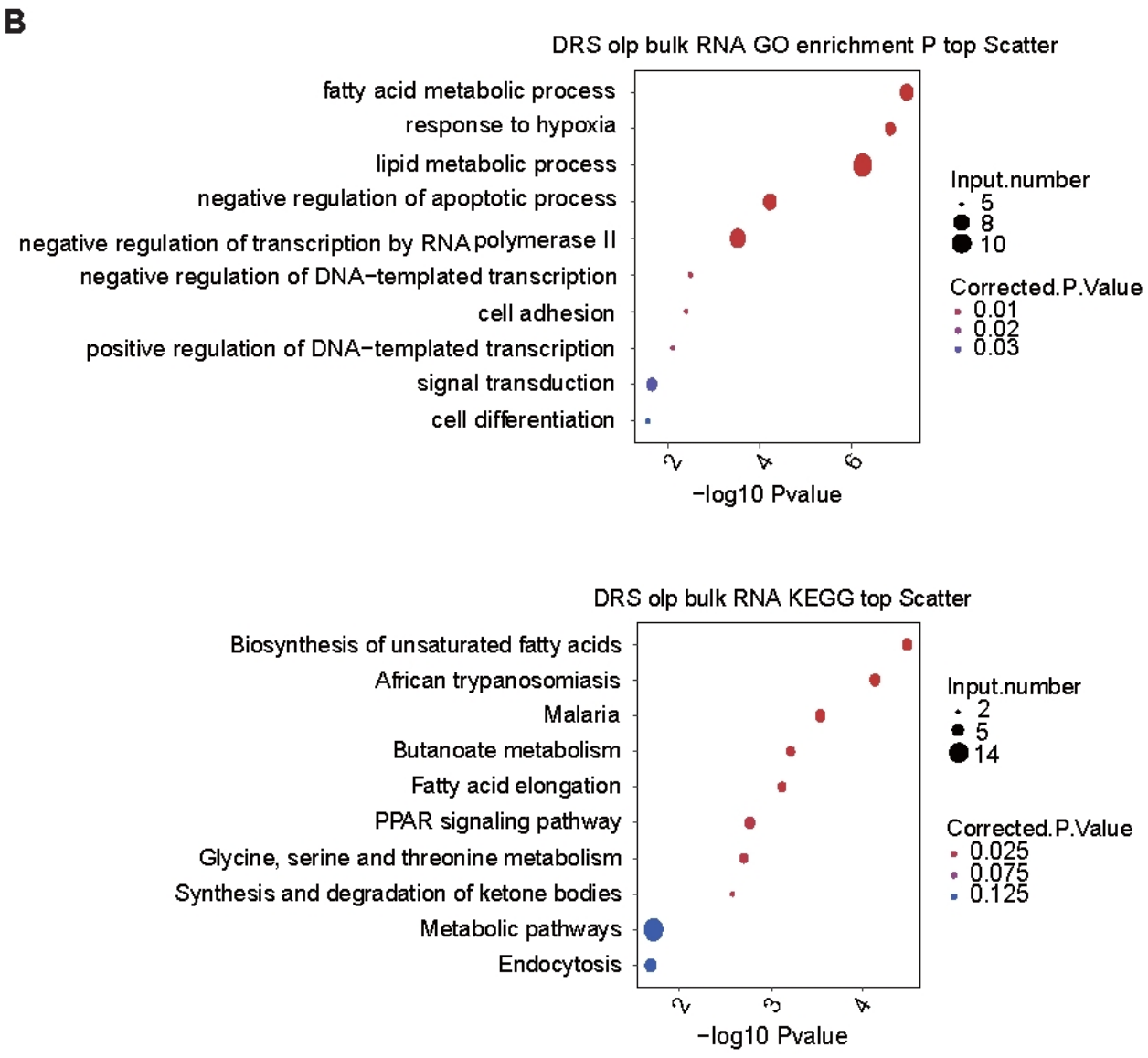

Figure S3. Enrichment analysis of differentially expressed genes/transcripts.

A. The Scatter plot exhibiting the most enriched GO biological process results of the known DETs.

B. GO and KEGG enrichment analysis of the intersected DEGs identified by both DRS-seq and bulk RNA-seq.

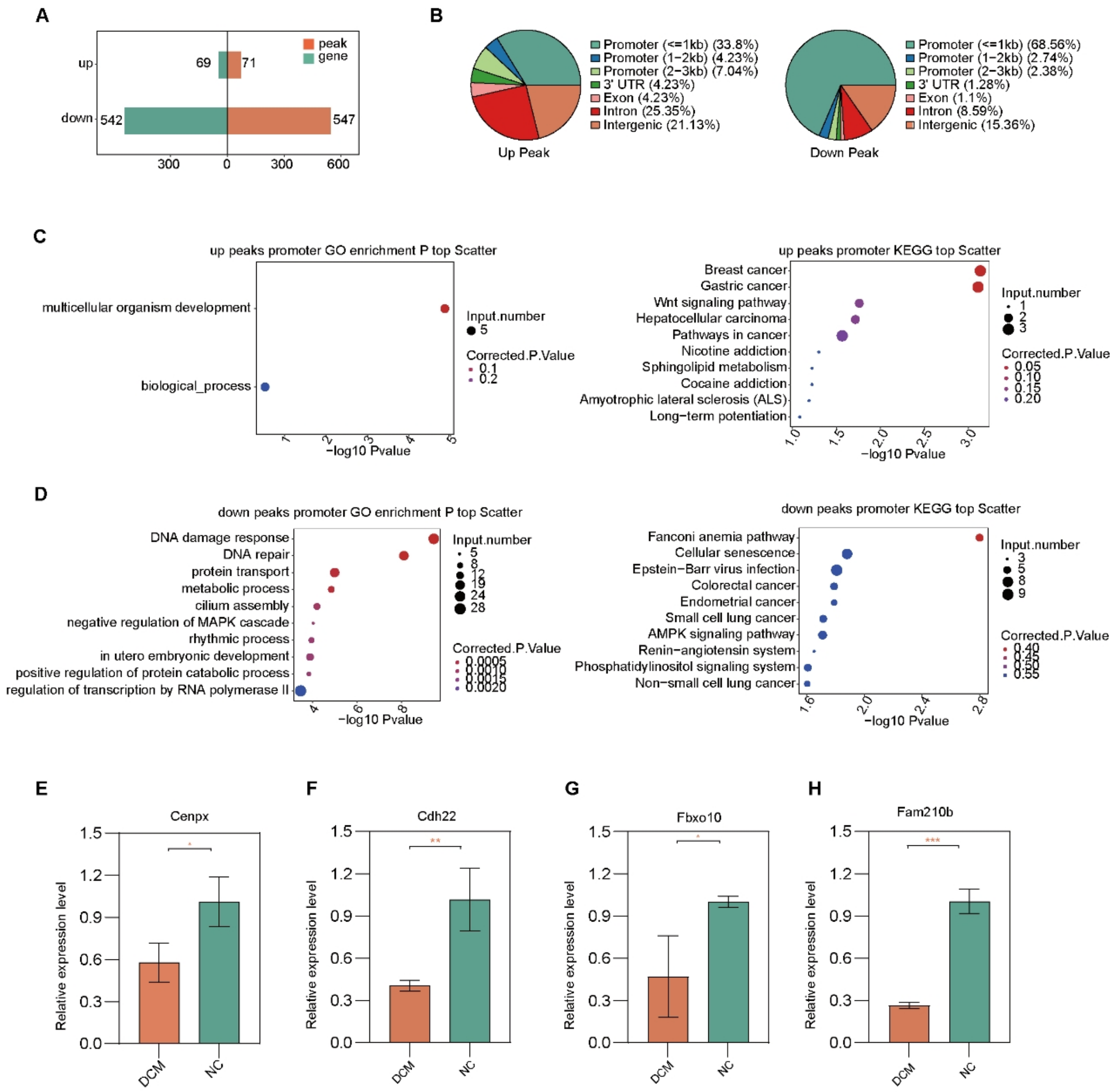

Figure S4. Genome-wide identification of differential chromatin accessibility peaks via ATAC-seq

A. The bar showing the number of differentially peak(Right panel) and the number of genes(Left panel) where they occur.

B. Pie chart showing the genomic distribution of differentially peaks.

C. GO/KEGG enrichment analysis of genes corresponding to promoter up_peaks.

D. GO/KEGG enrichment analysis of genes corresponding to promoter down_peaks.

F-H. The validation of expression level of *Cenpx, Cdh22, Fbxo10, Fam210b* in DCM and NC groups by RT-qPCR, with n = 3 per group. Error bars represent mean ± SEM, ***P-value < 0.001 , **P-value < 0.01, *P < 0.05.

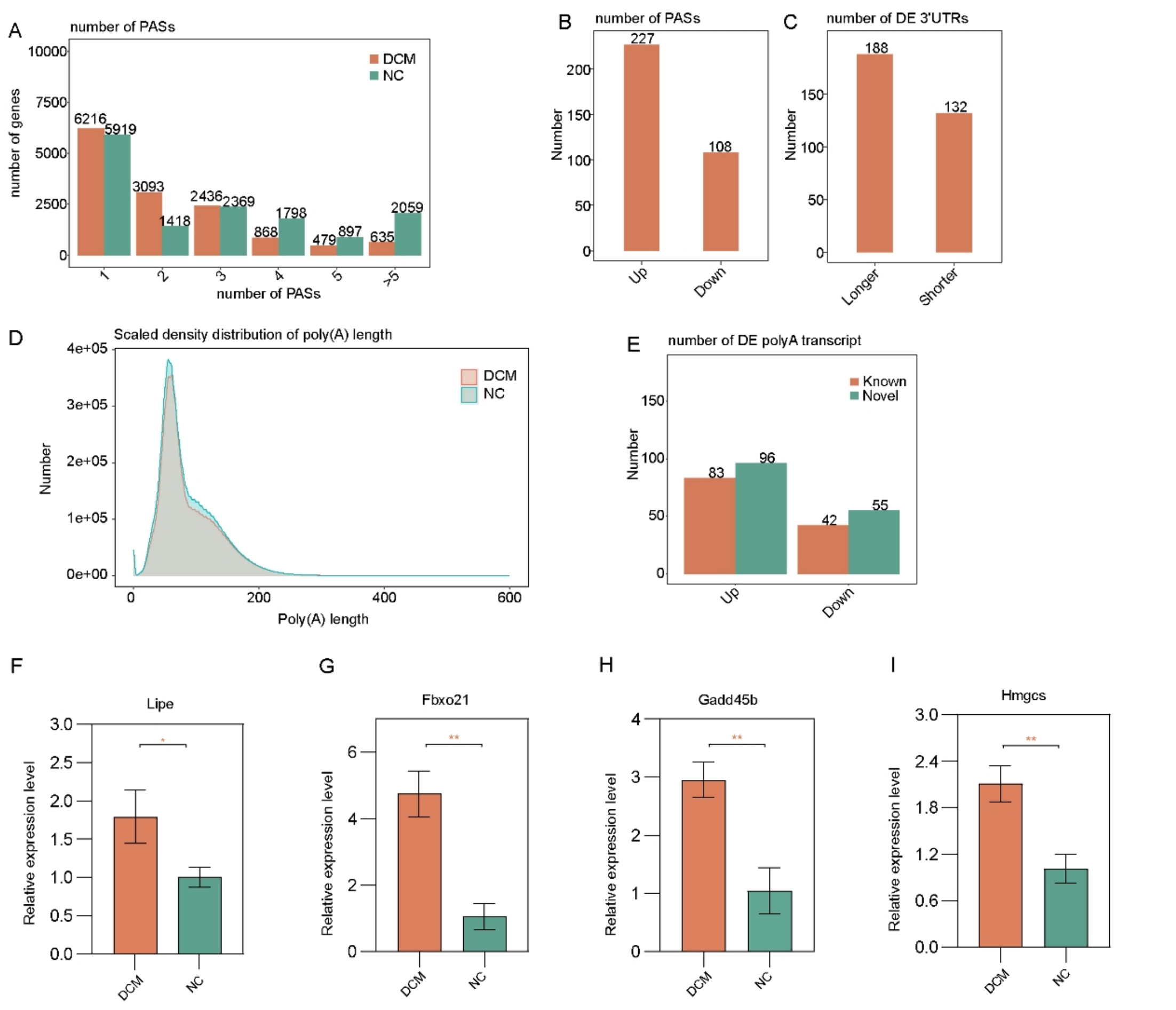

Figure S5. Polyadenylation site usage and 3' UTR dynamics in diabetic cardiomyopathy revealed by DRS-seq.

A. The bar showing the number of the APA sites detected in each genes.

B. The bar showing the number of the differentially APA sites.

C. The bar showing the number of the differentially length of 3'UTR.

D. Statistical analysis of polyA length distribution.

E. The bar showing the number transcript of the differentially length of polyA.

F-I. The validation of expression level of *Fbxo21, Gadd45b, Hmgcs2, Lipe* in DCM and NC groups by RT-qPCR, with n = 3 per group. Error bars represent mean ± SEM, **P-value < 0.01, *P < 0.05.
